# The Chikungunya virus: A reemerging cause of acute febrile illness in the high jungle of northern Peru

**DOI:** 10.1371/journal.pntd.0011787

**Published:** 2023-12-11

**Authors:** Miguel Angel Aguilar-Luis, Hugh Watson, Yordi Tarazona-Castro, Lucinda Troyes-Rivera, Felipe Cabellos-Altamirano, Wilmer Silva-Caso, Ronald Aquino-Ortega, Hugo Carrillo-Ng, Victor Zavaleta-Gavidia, Juana del Valle-Mendoza

**Affiliations:** 1 School of Medicine, Research Center of the Faculty of Health Sciences, Universidad Peruana de Ciencias Aplicadas, Lima, Peru; 2 Antiviral Research Unit, Evotec ID, Lyon, France; 3 Subregional Health Directorate of Jaen, Ministry of Health, Cajamarca, Peru; 4 Regional Laboratory of Cajamarca, Regional Health Directorate (Dirección Regional de Salud, DIRESA) of Cajamarca, Cajamarca, Peru; Faculty of Science, Ain Shams University (ASU), EGYPT

## Abstract

**Background:**

The Chikungunya virus (CHIKV) is an emerging arthropod-borne virus (arbovirus) that causes undifferentiated acute febrile illness. Cases of CHIKV may be under-reported in Peru, given the various difficulties in diagnosing it, such as lack of diagnostic tests in remote areas, the passive nature of epidemiological surveillance, and co-circulation of other arthropod-borne pathogens. Therefore, a study was conducted in the high jungle of northern Peru to determine the prevalence of CHIKV among febrile patients and describe their clinical characteristics.

**Methods:**

A cross-sectional study was conducted in the province of Jaen, Cajamarca, located in the high jungle of northern Peru. Patients attending primary healthcare centers within Cajamarca’s Regional Health Directorate were enrolled. The study took place from June 2020 through June 2021. Patients were eligible if they sought outpatient healthcare for a clinical diagnosis of acute febrile illness (AFI). Serum samples were collected from all patients, and the diagnosis of CHIKV was determined using real-time RT-PCR, as well as the detection of IgM antibodies by ELISA. A logistic regression model was employed to identify the risk factors for CHIKV, and the odds ratios (ORs) were calculated, along with their corresponding 95% confidence intervals (95% CI).

**Results:**

A total of 1 047 patients with AFI were included during the study period. CHIKV was identified in 130 patients of 1 047 (12.4%). Among the CHIKV positive cases, 84 of 130 (64.6%) were diagnosed by RT-PCR, 42 of 130 (32.3%) by IgM ELISA detection, and 4 of 130 (3.1%) by both assays. The majority of patients with CHIKV infection fell within the 18–39 years age group (50.0%), followed by the 40–59 years age group (23.9%) and those with 60 years or older (10.8%). The most common clinical symptoms observed in patients with CHIKV infection were headache (85.4%), myalgias (72.3%), and arthralgias (64.6%). The highest number of positive CHIKV cases occurred in May (23.1%), followed by March (20.0%) and February (13.8%) of 2021.

**Conclusion:**

The study reports a considerable frequency of CHIKV infections among patients with AFI from the high jungle of northern Peru. These findings highlight the importance of recognizing CHIKV as an ongoing pathogen with continuous transmission in various areas of Peru. It is crucial to enhance epidemiological surveillance by implementing reliable diagnostic techniques, as the clinical symptoms of CHIKV infection can be nonspecific.

## Author summary

The Chikungunya virus (CHIKV) has been one of the most challenging pathogens to affect humanity in recent years. Since its first major outbreak in 2004 in Africa, this virus has caused a series of epidemics worldwide, leading the scientific community and health systems to face a threat to public health. The authors conducted a study in the northern jungle of Peru to better understand the CHIKV situation. They determined that the most frequently infected were those in the age group of 18 to 39 years and the most common symptoms were headache, muscle aches and joint pain. It is highlighted that making an etiological diagnosis can be difficult in some remote areas, and that the clinical presentation overlaps with other mosquito-borne diseases. This research emphasizes the importance of epidemiological surveillance and scientific research in disease control, particularly acute febrile CHIKV illness.

## Introduction

The Chikungunya virus (CHIKV) is an arthropod-borne virus (arbovirus) classified under the *Alphavirus* genus. It is primarily transmitted by mosquitoes of the *Aedes* species, including *Aedes aegypti* and *Aedes albopictus* [1]. This pathogen is the etiological agent of the Chikungunya fever (CHIKF), which is often presented as an undifferentiated acute febrile illness (AFI) [2]. Although mortality rates of this disease are low, approximately 30% of the infected individuals develop a chronic disease that is characterized by persistent joint pain, tenosynovitis, and incapacitating polyarthralgia and can negatively affect the individual’s quality of life [1].

Since its reemergence in 2004, CHIKV has posed a global public health threat. It has caused cyclical epidemics in various regions, which were characterized by sporadic outbreaks interspersed with periods of epidemiological quietude ranging from months to years [3,4]. This epidemiological profile is caused by different factors related to environmental variables, climate change, mosquito ecology, viral genetics, human behavior, and susceptibility to infections in humans and vectors [3–5]. In year 2013, CHIKV arrived at the American continent and spread through the Caribbean [6]. The virus reached Brazil in 2014 [7] and expanded to other countries in South America.

Peru was no exception and the virus rapidly expanded throughout the country, as it was facilitated by the circulation of *Ae*. *aegypti* vector in different regions of this country. The first case of autochthonous transmission in Peru was reported in 2015 [1] and, since then, the virus has been circulating throughout the Peruvian territory. To date, previous studies in Andean and jungle regions of Peru reported variable rates of CHIKV prevalence in patients with acute febrile illness that range from 2.4% to 9.4% [8–10]. Moreover, in one study, CHIKV was the predominant etiological agent of AFI, surpassing the dengue cases [10].

The diagnosis of CHIKV may be troublesome, particularly in areas where other important arboviruses circulate. A rigorous epidemiological surveillance and differential diagnosis strategy are required in some regions of South America due to the co-circulation of CHIKV with other arboviruses, such as dengue virus (DENV), Zika virus (ZIKV), and Mayaro virus (MAYV) [3]. Cases of CHIKV may be under-reported in Peru, as its diagnosis undergoes a variety of difficulties, such as lack of access to diagnostic tests in remote areas, passive characteristics of the epidemiological surveillance, and co-circulation of other arthropod-borne pathogens [11]. Therefore, a study was conducted in the high jungle of northern Peru to identify the prevalence of CHIKV in febrile patients and describe their clinical characteristics.

## Materials and methods

### Ethics statement

This study was approved by the Research Ethics Board of the *Universidad Peruana de Ciencias Aplicadas* and the *Dirección Regional de Salud de Cajamarca* in Peru. Samples were collected within the framework of the epidemiological surveillance program of acute febrile syndrome in the Cajamarca region. According to the international ethical guidelines for health-related research involving humans that CIOMS and WHO prepared, this study does not require informed consent from individuals [12].

### Study location and patients

A cross-sectional study was conducted in the province of Jaen, Cajamarca, located in the high jungle of northern Peru. According to the national census in 2017, Jaen has a population of approximately 185 432 inhabitants, 52% of which live in urban areas and 48%, in rural areas [13]. This province is located at a mean altitude of 729 masl and has warm tropical weather with an annual mean temperature of 24.2°C. Several pathogens responsible for AFI have been reported in this region, which include DENV, Oropouche virus (OROV), ZIKV, leptospirosis, bartonelosis, rickettsia, and malaria, among others [8,9,14].

Patients attending primary healthcare centers of the Regional Health Directorate of Cajamarca were enrolled. This study was carried out as an aid for the national syndromic surveillance system for the diagnosis of arboviruses in febrile patients during the period between June 2020 and June 2021. Patients were included if they attended outpatient healthcare centers with a clinical diagnosis of acute febrile illness (AFI), which is defined as a temperature greater than 38°C for  less than 7 days without an identifiable source of infection and is related to one or more of the following signs and symptoms: headache, myalgia, arthralgia, retro-ocular pain, lower back pain, rash, and nausea, among others. The exclusion criteria were patients with an incomplete medical record, as well as patients with an identifiable source of infection, such as acute upper respiratory tract infections, pneumonia, and urinary tract infections.

### Samples

All patients underwent serum sample collection using the Vacuette TUBE Serum Separator Clot Activator (Vacuette; Greiner Bio-One, Kremsmünster, Austria). All samples were stored at − 80°C after collection and transported to Lima, Peru, to undergo molecular assays.

### Real-time RT-PCR assay for the detection of CHIKV

RNA extraction was performed from 200 μL of serum samples, and it was conducted with the High Pure RNA Isolation Kit (Roche Applied Science, Mannheim, Germany) according to the manufacturer’s instructions.

Amplification by real-time RT-PCR assay for CHIKV was performed with the primers and probe that Panning M. et al. described [15]. Sánchez-Carbonel et al. [10] describe the PCR conditions that are used in the study.

### ELISA for the detection of IgM antibodies against CHIKV

The presence of CHIKV IgM antibodies were also detected using Euroimmun ELISA (Euroimmun AG, Lübeck, Germany). Each serum sample was run in duplicate in accordance with the manufacturer’s instructions.

### Case definitions

Both assays were used to test all patients for the presence of CHIKV. Patients were considered positive for CHIKV infection if they had a positive RT-PCR test and/or a positive IgM ELISA, in addition to compatible clinical symptoms, according to the national guidelines for etiological diagnosis of acute febrile illness [16].

### Statistical analysis

All the data and information were compiled in a database using the Excel software from 2021 Microsoft 365. Data were analyzed using version 15.1 of Stata statistical software for Windows (StataCorp, College Station, TX, USA). Categorical variables were summarized using percentages, and continuous variables were shown as means ± standard deviation (SD). Variables associated with the development of CHIKV were determined using logistic regression analysis. The univariate and multivariate analyses were carried out to analyze the risk factors between CHIKV positives and negatives cases (binary logistic regression). The multivariate analysis included variables with a *p*-value less than 0.2 in the univariate analysis. A two-tailed *p*-value < 0.05 was considered statistically significant.

Maps were created using version 3.26.0 of the QGIS “Buenos Aires” software. The satellite map of Jaen was created at the Environmental Systems Research Institute (ESRI), and the maps of Peru and South American were acquired from the National Institute of Statistics and Computer Science (Instituto Nacional de Estadística e Informática, INEI) [https://www.inei.gob.pe/].

## Results

**Fig 1:**
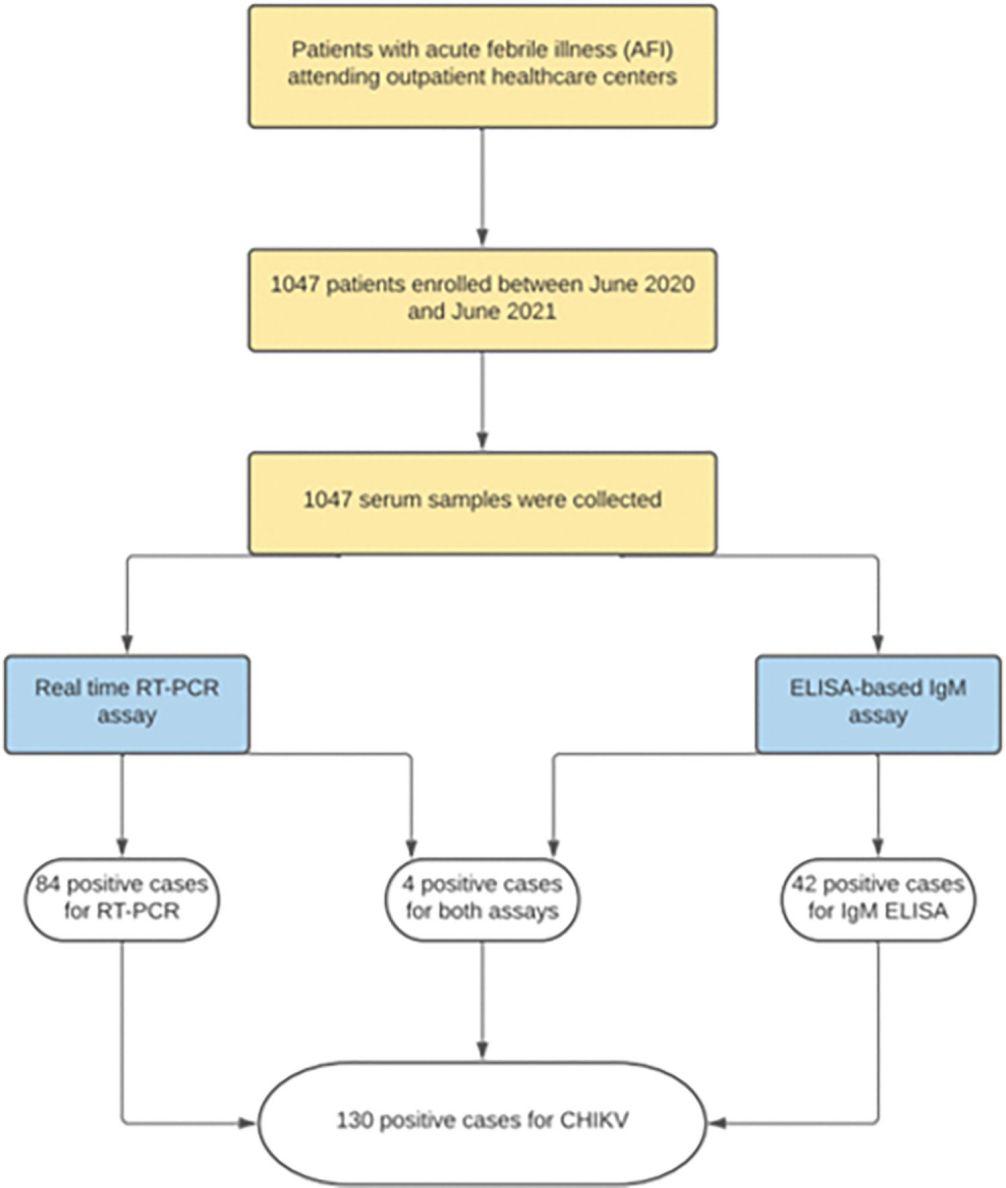
Research method and study design flowchart.

A total of 1 047 patients with AFI were enrolled between June 2020 and June 2021. Fig 1 represents a flowchart of the study design and study findings. Collection and testing of samples occurred between days 1 and 31 of illness. Patients were recruited and their samples were collected within 7 days from the onset of symptoms, and a maximum of 31 days was considered for the analysis of the collected sample. The identification of CHIKV was conducted in 130 of 1 047 patients (12.4%), 84 of which (64.6%) were diagnosed using RT-PCR, 42 (32.3%) through IgM ELISA detection, and 4 (3.1%) using both assays. The demographic characteristics of patients are shown in Table 1. Most patients with CHIKV infection corresponded to the 18–39 years age group (44.5%), followed by the 40–59 years age group (29.7%) and those with 60 years or older (13.5%). Furthermore, a greater proportion of female patients was identified with CHIKV (55.6%). On a binary logistic regression and in comparison, with patients without comorbidity, obese patients had 3.2 times higher odds of having a CHIKV infection (OR: 3.15, 95% CI: 1.06–9.33, *p* = 0.038), while the presence or absence of other demographic and clinical characteristics did not significantly affect the risk of CHIKV infection (Table 1).

**Table 1:** Demographical and clinical characteristics of patients with Chikungunya infection.

Characteristics	Total cases n = 1047 (%)	CHIKV negatives n = 917 (%)	CHIKV positives n = 130 (%)	Univariate analysis		Multivariate Analysis	
				OR (95% CI)	*p*-value	OR (95% CI)	*p-*value
							
**Age (years)**, mean ± SD	38.3 ± 18	36.7 ± 17	38.3 ± 18	2.26 (0.45–11.32)	0.323		
**Age group (years)**							
< 5	2 (0.2)	2 (0.2)	0 (0.0)	Ref			
5–11	41 (3.9)	34 (3.7)	7 (5.4)	1.87 (0.70–4.99)	0.203	1.56 (0.57–4.29)	0.391
12–17	61 (5.8)	54 (5.9)	7 (5.4)	1.18 (0.45–3.08)	0.741	1.06 (0.39–2.84)	0.912
18–39	466 (44.5)	401 (43.7)	65 (50.0)	1.47 (0.80–2.71)	0.206	1.32 (0.69–2.51)	0.405
40–59	311 (29.7)	280 (30.5)	31 (23.9)	1.00 (0.52–1.95)	0.990	0.95 (0.48–1.87)	0.887
≥ 60	141 (13.5)	127 (13.9)	14 (10.8)	∞	∞		
Unknown	25 (2.4)	19 (2.1)	6 (4.6)				
**Gender**							
Female	582 (55.6)	509 (55.5)	73 (56.2)	Ref.			
Male	461 (44.0)	404 (44.1)	57 (43.9)	0.98 (0.68–1.42)	0.931		
Unknown	4 (0.4)	4 (0.4)	-				
**Yellow fever vaccine**							
No	845 (80.7)	746 (81.4)	99 (76.2)	Ref.			
Yes	176 (16.8)	151 (16.5)	25 (19.2)	1.25 (0.78–2.00)	0.359		
Unknown	26 (2.5)	20 (2.2)	6 (4.6)				
**Comorbidities**							
None	839 (80.1)	731 (79.7)	108 (83.1)	Ref.			
Diabetes	77 (7.4)	72 (7.9)	5 (3.9)	0.47 (0.19–1.19)	0.111	0.54 (0.21–1.40)	0.207
Hypertension	33 (3.2)	30 (3.3)	3 (2.3)	0.68 (0.20–2.26)	0.525	0.81 (0.24–2.82)	0.746
Diabetes & Hypertension	15 (1.4)	14 (1.5)	1 (0.8)	0.48 (0.06–3.71)	0.485	0.57 (0.07–4.45)	0.590
Obesity	16 (1.5)	11 (1.2)	5 (3.9)	**3.08 (1.05–9.03)**	**0.041**	**3.15 (1.06–9.33)**	**0.038**
Asthma	8 (0.8)	6 (0.7)	2 (1.5)	2.26 (0.45–11.32)	0.323	2.43 (0.48–12.30)	0.283
Others	18 (1.7)	18 (2.0)	-	∞	∞		
Unknown	41 (3.9)	35 (3.8)	6 (4.6)				
**Days of symptoms**, mean ± SD	5.13 ± 6.2	5.22 ± 6.3	4.4 ± 5.4	0.98 (0.94–1.01)	0.192		

Data are represented as means ± standard deviation (SD) for continuous variables, and number (%) for categorical data. Unknown: No data for this group/category. Univariate and multivariable analysis using logistic regression, significant *p*-values are highlighted in bold (*p*-value < 0.05).^∞^: Omitted.

Table 2 shows the most common clinical symptoms among patients with positive and negative CHIKV detection. The most common clinical symptoms in the entire study population were headache (86.0%), myalgias (71.3%), and arthralgias (64.9%). As shown in Table 2, univariate and multivariate logistic regression analyses were performed to identify factors associated with CHIKV. Univariate analysis revealed that vomiting was significantly associated with CHIKV. In the multivariate analysis, only vomiting (OR: 1.64, 95% CI: 1.07–2.52, *p* = 0.022) was significantly associated with the presence of CHIKV. No severe hemorrhagic cases were identified among patients with CHIKV infection.

**Fig 2:**
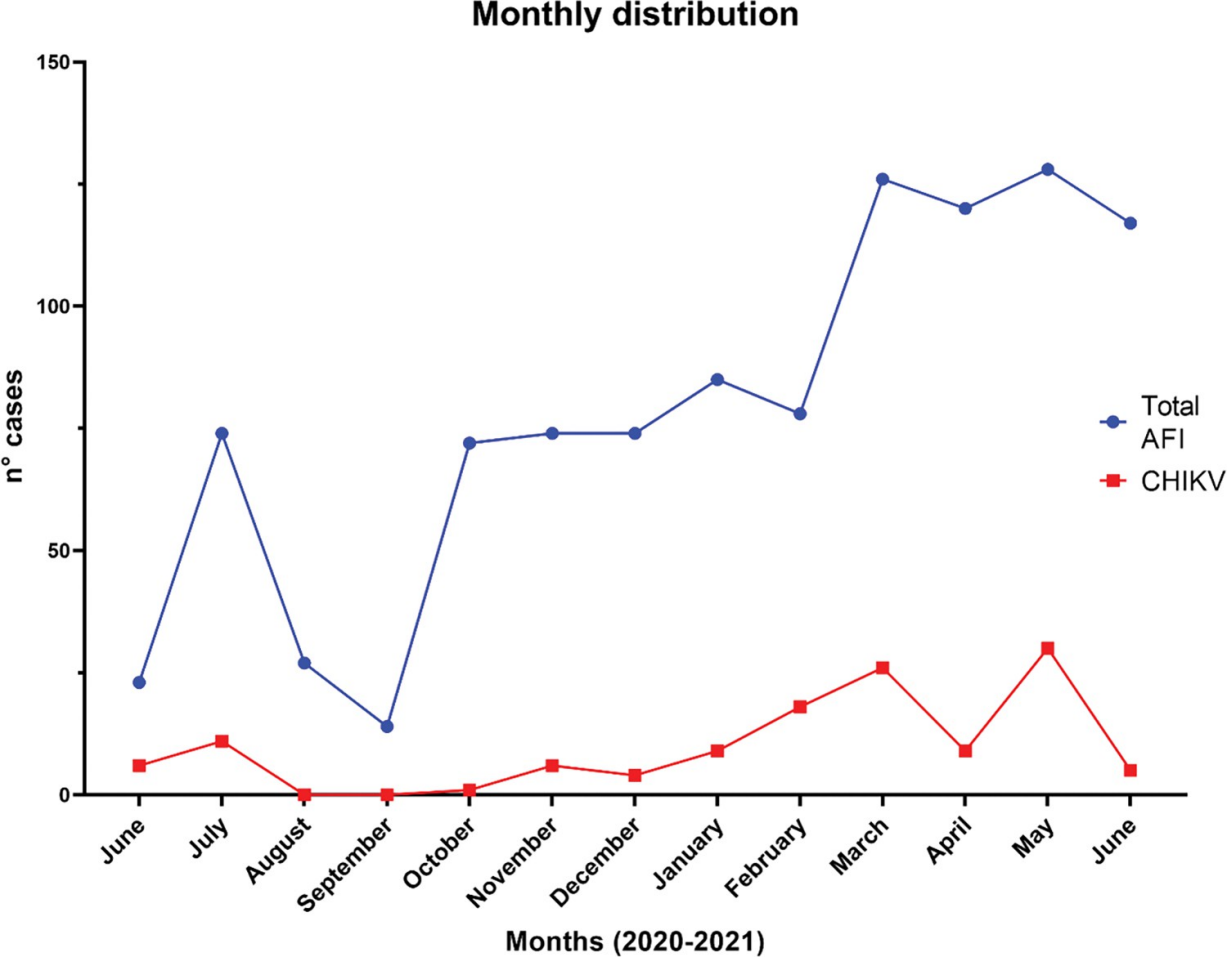
Monthly distribution of cases according to the pattern of infection.

**Fig 3:**
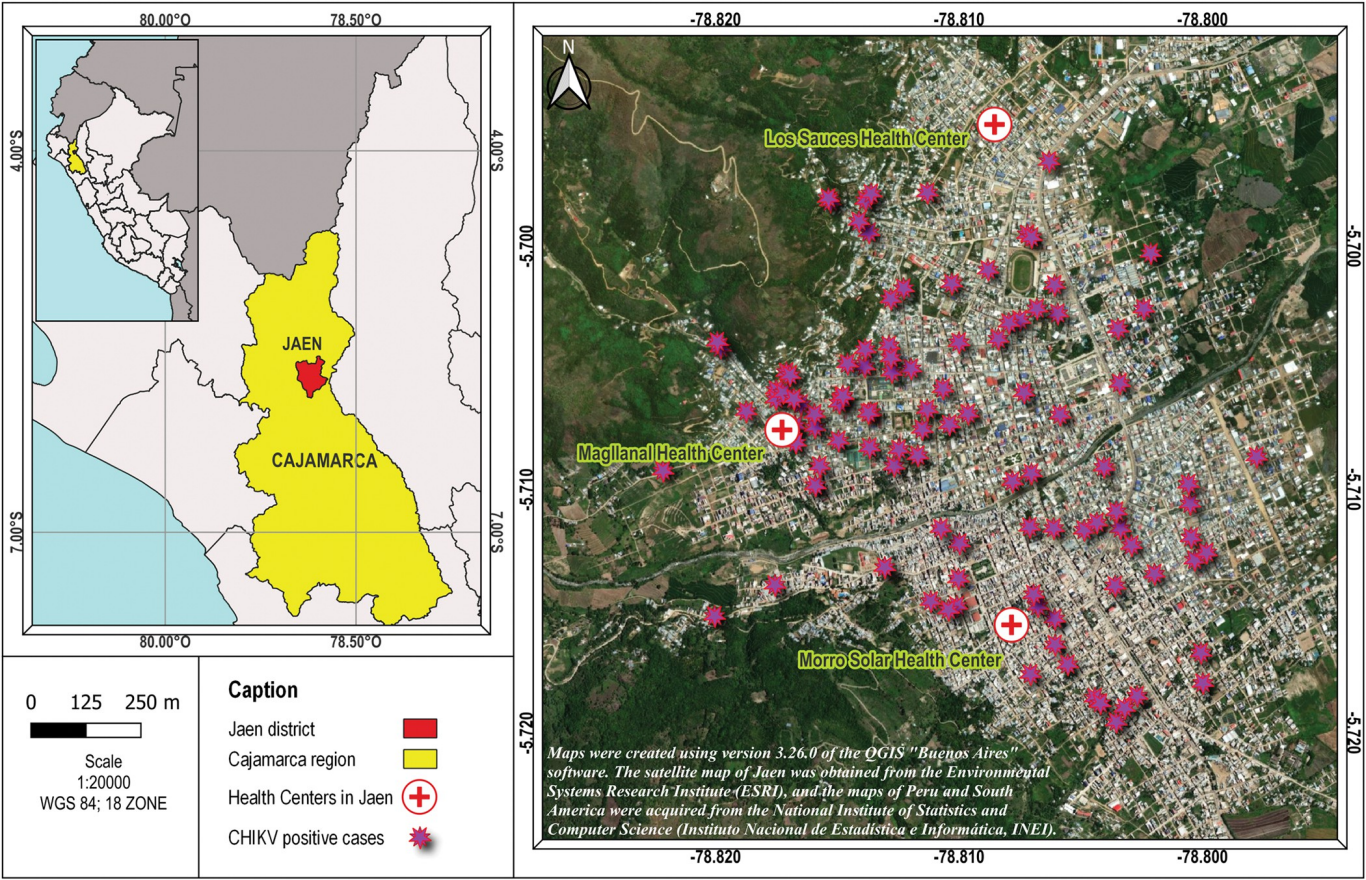
Geographical distribution of CHIKV positive cases in the district of Jaen, Peru. Maps were created using version 3.26.0 of the QGIS “Buenos Aires” software. The satellite map of Jaen was created at the Environmental Systems Research Institute (ESRI), and the maps of Peru and South American were acquired from the National Institute of Statistics and Computer Science (Instituto Nacional de Estadística e Informática, INEI) [https://www.inei.gob.pe/].

The monthly distribution of all AFI cases and CHIKV positive cases was evaluated and is shown in Figs 2 and 3. It can be observed that the majority of positive CHIKV cases occurred during the months of May (23.1%), March (20.0%) and February (13.8%) of 2021.

**Table 2:** Clinical signs and symptoms of patients with Chikungunya infection.

	Total cases n = 1047 (%)	CHIKV positives n = 130 (%)	CHIKV negatives n = 917 (%)	Univariate associations		Multivariate associations	
				OR (95% CI)	*p*-value	OR (95% CI)	*p-*value
**Clinical symptoms**							
Headache	900 (86.0)	111 (85.4)	789 (86.0)	0.95 (0.56–1.60)	0.840		
Myalgia	751 (71.3)	94 (72.3)	657 (71.6)	1.03 (0.69–1.56)	0.876		
Arthralgia	680 (64.9)	84 (64.6)	596 (65.0)	0.98 (0.67–1.44)	0.932		
Fever at enrollment	627 (59.9)	69 (53.1)	558 (60.8)	0.73 (0.50–1.05)	0.091	0.71 (0.49–1.03)	0.068
Retro-ocular pain	481 (45.9)	60 (46.1)	421 (45.9)	1.01 (0.70–1.46)	0.958		
Lumbar pain	407 (38.9)	42 (32.3)	365 (39.8)	0.72 (0.49–1.07)	0.102	0.75 (0.50–1.10)	0.143
Nausea	277 (26.5)	37 (28.5)	240 (26.2)	1.12 (0.75–1.69)	0.580		
Hand polyarthralgia	247 (23.6)	33 (25.4)	214 (23.3)	0.24 (1.71–0.00)	0.732		
Foot polyarthralgia	217 (20.7)	30 (23.1)	187 (20.4)	0.26 (1.82–0.00)	0.755		
Vomiting	205 (19.6)	35 (26.9)	170 (18.5)	1.62 (1.06–2.47)	**0.025**	1.64 (1.07–2.52)	**0.022**
Arthritis	150 (14.3)	17 (13.1)	133 (14.5)	0.89 (0.52–1.52)	0.664		
Rash	134 (12.8)	19 (14.6)	115 (12.5)	1.19 (0.71–2.02)	0.508		
Conjunctivitis	57 (5.4)	5 (3.8)	52 (5.7)	0.67 (0.26–1.70)	0.394		
Sore throat	3 (0.3)	0 (0.0)	3 (0.3)	∞	∞		
**Warning signs**							
Persistent abdominal pain	7 (0.7)	1 (0.8)	6 (0.6)	1.18 (0.14–9.86)	0.881		
Chest pain	3 (0.3)	0 (0.0)	3 (0.3)	∞	∞		
Persistent vomiting	1 (0.1)	0 (0.0)	1 (0.1)	∞	∞		
Tachycardia	1 (0.1)	0 (0.0)	1 (0.1)	∞	∞		
BPD (<20 mmHg)	1 (0.1)	0 (0.0)	1 (0.1)	∞	∞		

OR = odds ratio; CI = confidence interval; BPD: blood pressure differential, ^∞^: omitted because of collinearity and observations are insufficient. Univariate and multivariable analysis using logistic regression, significant *p*-values are highlighted in bold (*p*-value < 0.05).

## Discussion

The Chikungunya virus (CHIKV) is considered an emerging pathogen that causes acute febrile illness (AFI) in many regions of Peru [8]. However, the prevalence of this pathogen may be underestimated due to its clinical similarity with other arboviral infections, such as DENV and ZIKV, which can circulate in the same geographical regions and are transmitted by the same vector [17]. One of the main limitations in these areas is the lack of access to reliable diagnostic tests, such as molecular and serological techniques; therefore, a study was conducted to evaluate the prevalence of CHIKV in febrile patients from the high jungle of the northern area of Peru.

It is reported a prevalence of 12.4% (130/1 047) among the total febrile patients enrolled during the 1-year study period. The frequency of CHIKV-positive tests in the different age groups was proportional to the number of AFI cases seen and broadly corresponded to the general age distribution in the adult Peruvian population. The slight predominance of female patients may be attributed to the endophilic feeding habits of the *Aedes* mosquitoes [18]. The prevalence reported in this study is greater than in previous studies that evaluated the presence of CHIKV in Peru, and the similarity in the incidence that was observed in the different age groups is consistent with a lack of previous exposure to CHIKV and a consequent lack of immunity in the population. For example, Sánchez-Carbonel et al. [10] evaluated the prevalence of DENV, ZIKV and CHIKV in patients with AFI from the Peruvian coast. They reported that among 496 febrile patients, 34.3% were positive for DENV, 7.9% for ZIKV, and 4.6% for CHIKV. Another study in the Peruvian amazon basin [8] reported that CHIKV was the most frequent arbovirus among 139 febrile patients with 9.4%, which represented more cases than OROV, ZIKV and DENV. Studies from other countries in Latin America report differing frequencies of this pathogen; for example, Lima et al. [17] reported a prevalence of 3.84% of CHIKV cases among 182 patients, while a study in Puerto Rico that was conducted during a 3-year study period found that CHIKV corresponded to the 18.2% of the febrile patients [19]. These differences are related to various factors, among which are the seasonality on which the volume of rainfall and temperature depend, and the use of serological techniques for diagnosis, since antibodies are detectable for a longer time [17]. It should be mentioned that the prevalence of CHIKV is heterogeneous among the different Latin American countries and even in different regions within the same country; therefore, molecular diagnosis is critical for the epidemiological surveillance of this virus [1,20].

For the current study, it was used two different diagnostic tests to identify CHIKV infections: the identification of viral RNA by RT-PCR and the detection of IgM antibodies by ELISA. We could highlight that both assays are reliable techniques for the diagnosis of this virus and are recommended by the Peruvian guidelines for the diagnosis of arboviral diseases [15]. On the other hand, the CDC recommends the use of real-time RT-PCR in samples collected within 6 days since the onset of the disease, as well as the use of serological tests to identify IgM in samples with more than 6 days after the disease onset [21]. The rationale behind this is that CHIKV rapidly replicates in the infected host, and viral RNA can be detected by real-time RT-PCR during the first week since the disease onset [18,19]. Moreover, IgM antibodies typically develop after the first days of disease and become detectable in serum from days 4 to 7 after illness onset [21]. The patients included in the current study were recruited within the first 7 days since disease onset, and, therefore, the application of these two diagnostic tests may be adequate to identify most positive cases. Although both diagnostic tests have optimal diagnostic performance, they are not perfect. Several limitations have been reported for both tests; for example, real-time RT-PCR continues to be the main test for confirming Chikungunya infection, but it requires certain laboratory equipment that may not be available in rural areas and is also an expensive test that requires trained laboratory staff [11]. Regarding the limitations of serological tests, they may have cross-reactivity to other alphaviruses and with DENV [22–24] and have reported long term persistence of IgM antibodies after the infection has cleared [25].

The most common clinical signs and symptoms in patients with positive CHIKV infection were described. It has been reported that most cases of Chikungunya fever present as a self-limiting mild febrile disease, that characterizes by debilitating arthralgias that may persist for months [26]. It is also reported in this study that the most common symptoms CHIKV patients presented were headaches, myalgias, and arthralgias. However, all symptoms evaluated were similar in patients with positive and negative CHIKV infection. In fact, other studies have also reported that it may be difficult to differentiate CHIKV infections if they only based on symptoms. For example, a study performed in Mexico also reported no significant differences in clinical presentations between those with CHIKV confirmed infection and those without CHIKV, myalgias, headaches and arthralgias being predominant among both groups [27]. In our study, the presence of vomiting was frequent and had statistical significance. This is consistent with other studies and reviews that indicate that vomiting is frequent in the clinical presentation of CHIKV infection without being a determinant clinical sign by itself [28,29].

The highest frequency of positive cases was recorded during the months of February, March, and May. These months are within the reported arbovirus transmission season, which is from January to May in the southern hemisphere. This transmission characteristic is due to the dynamics and distribution of the vector populations, as well as to the social and ecological factors of the region [30].

Nonetheless, significant efforts are being made to help clinicians differentiate CHIKV infections from other causes of AFI. For example, Alvarado et al. [19] determined clinical predictors to distinguish CHIKV from other causes of AFI and DENV infections. They reported that significant predictors for CHIKV were joint pain, muscle, bone or back pain, skin rash, and red conjunctiva. Likewise, Lee et al. [31] also reported that some clinical signs and symptoms may predict CHIKV infection such as myalgias and arthralgias. Although the epidemiological context may help to guide a clinical diagnosis, the overlapping clinical picture between the different pathogens responsible for AFI highlights the need for precise diagnostic tests for the identification of etiological agents.

Among the comorbidities studied, obese patients had a higher probability of having CHIKV infection. This result is reinforced by studies that describe this condition as a host risk factor for infection [32]. In addition to this, epidemiological evidence indicates that there is also an increased risk of serious complications of viral infection [33]. This is partly because adipose-derived cytokines and chemokines altered in obesity by chronic low-grade inflammation affect the immune response and make obese patients prone to severe disease or death [32,34].

Our study encountered some limitations. Firstly, past CHIKV infection was not evaluated because in outbreak situations such as this acute febrile illness, healthcare resources were overstretched and limited. Also, we did not consider testing for past CHIKV infections in all patients as it requires a significant amount of resources that was not included in the protocol.

In conclusion, we report a considerable frequency of CHIKV infections in patients with AFI from the high jungle of northern Peru. These findings should raise awareness that CHIKV is a pathogen with continuous transmission in different regions of Peru. Moreover, the clinical signs and symptoms of Chikungunya fever may be nonspecific and overlap with other causes of AFI; therefore, the implementation of reliable diagnostic methods, such as molecular and serological techniques, is crucial, particularly in regions where other emerging arboviruses circulate.
